# Cinquo-Quinine

**Published:** 1877-07-20

**Authors:** 


CINQUO QUININE.—This preparation is evidently growing in popularity, based, as we believe, not only upon faith in the reliability of the house that manufactures it, but upon actual merit in the article itself. The paper of Dr. King, of Atlanta, Ga., upon this subject, in the present issue, will repay perusal.

